# Modeling Human Frailty with a Smart Home-Based Approximation of Entropy

**DOI:** 10.1145/3584371.3613017

**Published:** 2023-10-04

**Authors:** Katherine A. Wuestney, Beiyu Lin, Diane J. Cook, Roschelle L. Fritz

**Affiliations:** Washington State University, Vancouver, Washington, USA; University of Nevada Las Vegas, Las Vegas, USA; Washington State University, Pullman, Washington, USA; Washington State University, Vancouver, Washington, USA

**Keywords:** behavior complexity, data complexity, frailty, entropy, smart homes, CGR/USM

## Abstract

The global population is aging. These older adults face changes in health that result in increasing frailty. Digital biomarkers created by passive, continuous sensors offer an early indicator of impending frailty that can be used to delay or reverse frailty. Building on the notion of a human as a complex system, we introduce and compare three methods to model and estimate the complexity of indoor human behavior. Each method offers potential benefits for estimating human frailty. We introduce a formalization of the approaches, extend their use for arbitrary-size sensor suites, and demonstrate how they can be used to visualize and calculate a person’s behavioral complexity based on smart home data collected continuously for an older adult subject.

## 1 INTRODUCTION

Aging involves an array of biopsychosocial changes which interact in multidimensional, dynamic pathways to produce a range of common symptom profiles, or syndromes, frequently experienced by older adults. There is a growing consensus among researchers that the heterogeneous trajectories of aging syndromes cannot be fully understood without a complex systems approach [5]. Such an approach views humans as complex systems with multiple levels of factors interacting in nonlinear ways. These complex dynamics result in the emergence of system properties, such as homeostasis or resilience, which are greater than the sum of the lower-scale factors. From a complex systems framework, loss of complexity in any part of the human system may lead to the same emergent outcomes of frailty and decline [12].

Mounting evidence suggests that the complexity of a person’s observable behavior signals (e.g. gait variability and standing postural sway among others) could serve as a ‘digital biomarker’ of the body’s level of frailty [5]. Digital biomarkers created passively via ubiquitous sensing technologies offer new opportunities for continuous, minimally obtrusive, and remotely delivered assessment of health conditions in ecologically valid real-world settings [4]. Frailty, a geriatric syndrome characterized by functional decline across multiple physiologic systems and reduced capacity to overcome internal and external stressors, can be conceptualized as a complex system on the brink of failure [15]. Measuring change of complexity of the human system’s signals would then be an indicator of how much complexity has been lost and, therefore, how close the system is to failure (i.e., frailty). This Loss of Complexity Hypothesis may explain why an individual with health deficits related to cardiovascular disease develops the same phenotypic frailty presentation as another individual with a diagnosis of COPD and no cardiovascular disease or another individual with mild deficits in a number of physiologic domains but no formal chronic disease diagnosis [8, 12].

The progression of frailty severity can be slowed if not altogether reversed, making early identification and intervention of worsening frailty a critical function of geriatric healthcare [9]. Compared to conventional health measures of frailty, which are not amenable to continuous measurement and can be time consuming, expensive, and physically invasive, digital biomarkers based on purely passively-collected smart home sensor data would further minimize patient burden and may extend the reach of community prevention and early intervention of frailty.

In this paper, we propose measuring the complexity of human indoor activity using smart home sensor data, defined as the level of randomness and irregularity of sensor transition patterns, as a potential digital biomarker for detecting change in a person’s frailty. The central hypothesis motivating this work is that the complexity of smart home movement trajectories of older adults diminishes with health decline and frailty. We introduce a novel approach to quantify and visualize the complexity of smart home data using universal sequence maps (USM) with the Rényi quadratic entropy measure (USM-Rényi) [18]. A USM is a bijective projection of a sequence from a discrete state space (e.g., sequence of sensor readings) to a real-valued state space. We expand the original formula for USM-Rényi to symbolic sequences of arbitrary alphabet size and compare its sensitivity to changes with two other entropy measures designed for short time series, Approximate Entropy and Sample Entropy, using Monte Carlo Simulations.

Our contributions include formalizations of behavior complexity measures and a new proof of USM-Rényi for arbitrary alphabet sizes. We demonstrate which entropy measure converges fastest and provides the narrowest error on short sample lengths. Additionally, we present a novel application of the USM algorithm to the visualization of smart home motion motifs at multiple scales. Our analysis demonstrates how these visualizations can easily discriminate days with and without novel complex activity (due to visitors and housekeeping) in a case example.

## 2 RELATED WORK

To date, few studies of digital biomarker technologies targeting frailty utilize an ambient sensor platform or passive monitoring, and none utilize a complex systems framework of aging [17]. Schütz et al. [2022] include entropy as one of 1,269 digital biomarkers they explored from smart home data for 45 older adults using a “holistic systems” machine learning approach to predict frailty among other outcomes. Of their markers, they found fridge entropy to be moderately negatively correlated (r=−0.25) with frailty. Because their entropy measure was based on the uncorrelated frequency of hourly readings, the approach misses higher-order behavior complexities such as transitions between locations.

Other smart home studies explore correlated entropy measures that do reflect these multi-order transitions. Unlike our study, these related analyses focus primarily on movement predictability, rather than health outcomes [19, 20]. Howedi et al. [2020] leveraged Sample Entropy and Approximate Entropy, two measures we analyze in this paper also, to identify periods of multi-occupancy in smart home data. In order to detect change in complexity of motion trajectories, we need measures which reflect multi-order complexity of motion trajectories that are reliable with relatively short time series. Of these studies, only Wang et al. [2023] explored the sensitivity of the measures to sequence length and none explored the sensitivity of the entropy measures to number of sensors (i.e., alphabet size), even though information entropy and its derivatives are known to be sensitive to both alphabet size and sequence length [7].

## 3 SMART HOME DATA

For our study, we analyze data collected by ambient sensors as part of the CASAS Smart Home in a Box [6]. As shown in Figure 1, sensors include motion detectors, magnetic door sensors, ambient light and temperature sensors. All sensors generate a reading whenever they detect a change in state (e.g., when motion starts or stops in a sensor’s field of view.) Each sensor reading includes a corresponding date, time, sensor identifier, and sensed state (ON/OFF for motion, OPEN/CLOSED for doors, numeric values for light and temperature sensors). As in [19, 20] we construct our motion trajectories using the ON and OPEN readings, excluding OFF and CLOSE.

## 4 METHODOLOGY

We model human behavior based on movement trajectories captured by smart home sensors. We assume that a person’s trajectory is a non-stationary stochastic process whose complexity changes over time in association with health status. We then can use the model to estimate the complexity of the underlying “human system” and the model is used as a basis for estimating a person’s frailty. Throughout the paper, we represent a smart home sensor reading trajectory as a symbolic sequence, X, whose alphabet $S=\left\{s_{1},\ldots ,s_{d}\right\}$ represents the set of sensors with cardinality d and $x_{i}$ represents the reading generated at index i in sequence X. In our methods, we implement and compare three methods for estimating complexity of smart home sensor sequences: USM, Approximate Entropy, and Sample Entropy. We compare the performance of each method on short symbolic sequences using Monte Carlo simulation and demonstrate the potential of USM methodology in a smart home case study.

### 4.1 Method 1: Universal Sequence Maps (USM)

Universal sequence mapping maps a sequence with a discrete alphabet to a real-valued state space in $R^{\text{d}}$. USMs function as generalized order-free Markov transition matrices of symbolic sequences (i.e., sequences of sensor readings). We compute an entropy estimate of a sequence X based on the density of USM coordinates.

Generating the USM space is based on the algorithm for chaos game representation (CGR, [11]). First, each symbol in a sequence is assigned to a vertex of a unit hypercube such that the vertex coordinates {Y} of the USM hypercube are equal to the rows of the d×d identity matrix, reflecting a one-hot encoding of S. Next, starting at the beginning of the sequence, the USM coordinate, $a_{i}$, for each symbol in X is computed iteratively as the midway point between the vertex associated with that symbol and the previous symbol’s coordinate. This is given by Equation 1, where $y_{i}$ is the vertex coordinate associated with the $i^{\text{th}}$ symbol in X.


(1) $$a_{i}=a_{i-1}+\frac{1}{2}\left(y_{i}-a_{i-1}\right),\;i=1,\ldots ,N$$

This approach ensures that the USM projection is always bijective, no matter the alphabet size [2]. Theoretically, this means that a single USM coordinate contains information of the entire sequence and conversely the entire sequence could be recovered from a single coordinate. USM is therefore a universal, non-parametric method for analyzing the statistical properties on multiple scales of symbolic sequences of any alphabet size. CGR/USM images, such as the one shown in Figure 2, reveal characteristic patterns related to the density of different k-grams in the sequence. This is because all coordinates within the same sub-quadrant will always share the same k preceding symbols. Therefore, the Euclidean distance between any two points in the USM does not indicate the nearness within the parent sequence but rather the length of a shared suffix. Because of this property, the USM map depicts all k-gram frequencies simultaneously. This makes the USM an *order free* generalization of a Markov Chain Transition Matrix, with the $k^{\text{th}}$ order transition frequencies equal to the density of coordinates within the sub-quadrants with side lengths $2^{-k}$ [1].

Based on this property, Vinga and Almeida proposed a method for measuring the continuous Rényi entropy of 4-dimensional USM (i.e., to analyze sequences with an alphabet of size 4) based on calculating the density distribution of a USM with a Parzen kernel density estimate with Gaussian kernel density function [18]. The equation for this 4-dimensional USM-Rényi is given in Equation 2, where $d_{ij}$ is the squared Euclidean distance between USM coordinates $a_{i}$ and $a_{j}$ and σ determines the size of the kernel.


(2) $$H_{2}(USM)=-\text{ln}\frac{1}{N^{2}}\sum_{i=1}^{N}\sum_{j=1}^{N}\frac{1}{16\pi^{2}\sigma^{4}}\text{exp}\left(-\frac{1}{4\sigma^{2}}d_{ij}\right)$$

We now offer a new proof that extends this Rényi equation for an alphabet of any size, d. Rényi entropy was introduced as a generalization of Shannon entropy and includes an order parameter α which determines the weighted contribution of improbable events to the overall entropy measure. The limit of Rényi entropy ${\text{lim}}_{\alpha \to 1}H_{\alpha }(X)$ is Shannon’s entropy measure. The formula for Rényi entropy of a continuous probability density function f(x) is:

(3) $$H_{\alpha }(X)=\frac{1}{1-\alpha }\text{ln}\int f^{\alpha }(x)dx$$

We first substitute the kernel density equation of a spherical Gaussian kernel [18] for f(x) in Equation 3:

(4) $$H_{2}(USM)=-\text{ln}\int {\left(\frac{1}{N}\sum_{i=1}^{N}\frac{1}{(2\pi {)}^{d/2}{\left|\sigma^{2}I_{d}\right|}^{1/2}}e^{\left(-\frac{1}{2}{\left(x-a_{i}\right)}^{T}{\left(\sigma^{2}I_{d}\right)}^{-1}\left(x-a_{i}\right)\right)}\right)}^{2}dx\;=-\text{ln}\int \frac{1}{N^{2}}\sum_{i=1}^{N}\sum_{j=1}^{N}\left(\frac{1}{(2\pi {)}^{d/2}{\left|\sigma^{2}I_{d}\right|}^{1/2}}e^{\left(-\frac{1}{2}{\left(x-a_{i}\right)}^{T}{\left(\sigma^{2}I_{d}\right)}^{-1}\left(x-a_{i}\right)\right)}\right)\times \left(\frac{1}{(2\pi {)}^{d/2}{\left|\sigma^{2}I_{d}\right|}^{1/2}}e^{\left(-\frac{1}{2}{\left(x-a_{j}\right)}^{T}{\left(\sigma^{2}I_{d}\right)}^{-1}\left(x-a_{j}\right)\right)}\right)dx$$

Applying the constant coefficient rule of integration and the summation rule of integration, we move the integral operator inside the summation. The entropy is now a convolution of the two Gaussians:

$$H_{2}(USM)=-\text{ln}\frac{1}{N^{2}}\sum_{i=1}^{N}\sum_{j=1}^{N}\frac{1}{(2\pi {)}^{d/2}{\left|\sigma^{2}I_{d}+\sigma^{2}I_{d}\right|}^{1/2}}\times e^{\left(-\frac{1}{2}{\left(a_{i}-a_{j}\right)}^{T}{\left(\sigma^{2}I_{d}+\sigma^{2}I_{d}\right)}^{-1}\left(a_{i}-a_{j}\right)\right)}$$

Because we are using a fixed kernel volume approach, $\sigma^{2}$ is held constant across all kernels, so we can further simplify $\sigma^{2}I_{d}+\sigma^{2}I_{d}$ to $2\sigma^{2}I_{d}$. Then ${\left|2\sigma^{2}I_{d}\right|}^{1/2}={\left(2^{d}\sigma^{2d}\right)}^{1/2}=2^{d/2}\sigma^{d}$ and ${\left(2\sigma^{2}I_{d}\right)}^{-1}=\frac{1}{2\sigma^{2}}I_{d}$. The former equality is due to the fact that the determinant of a d×d scalar matrix is the scalar constant raised to the $d^{\text{th}}$ power, $\left|cI_{d}\right|=c^{d}$. The latter is due to the fact that the inverse of a diagonal matrix is a diagonal matrix whose principal diagonal is made up of the reciprocals of the elements of the original matrix, which in this case is $\frac{1}{2\sigma^{2}}$. Plugging these two simplified terms into the entropy equation, we can perform further simplifications based on the properties of matrix-vector multiplication involving scalars and the identity matrix:

(5) $$H_{2}(USM)=-\text{ln}\frac{1}{N^{2}}\sum_{i=1}^{N}\sum_{j=1}^{N}\frac{1}{(2\pi {)}^{d/2}2^{d/2}\sigma^{d}}e^{\left(-\frac{1}{2}{\left(a_{i}-a_{j}\right)}^{T}\frac{1}{2\sigma^{2}}I_{d}\left(a_{i}-a_{j}\right)\right)}\;=-\text{ln}\frac{1}{N^{2}}\sum_{i=1}^{N}\sum_{j=1}^{N}\frac{1}{{\left(2\pi^{1/2}\sigma \right)}^{d}}e^{\left(-\frac{1}{4\sigma^{2}}{\left(a_{i}-a_{j}\right)}^{T}\left(a_{i}-a_{j}\right)\right)}$$

For u and vd-vectors, the product of the vector of the form (u−v) with its transpose $(u-v{)}^{T}$ is equivalent to the squared Euclidean distance between u and v. Let $d_{ij}$ represent the squared Euclidean distance between $a_{i}$ and $a_{j}$, then the entropy is rewritten as shown in Equation 6, which we observe is congruous with Equation 2 when d=4.


(6) $$H_{2}(USM)=-\text{ln}\frac{1}{N^{2}}\sum_{i=1}^{N}\sum_{j=1}^{N}\frac{1}{{\left(2\pi^{1/2}\sigma \right)}^{d}}e^{\left(-\frac{1}{4\sigma^{2}}d_{ij}\right)}$$

### 4.2 Method 2: Approximate Entropy (ApEn)

In addition to the USM derivation of entropy, we consider two other popular entropy methods for detecting change in complexity of time series which have previously been applied to smart home data. The first of these, Approximate Entropy, is commonly used to compare the complexity of short real-valued sequences. However, it has been shown to perform well for binary symbolic sequences and should in theory be appropriate for alphabets of any size [13].

ApEn requires two parameters to be defined, the embedding dimension m and tolerance r. Given a sequence X of length N and parameters m=2 and r=1, ApEn generates a set of vectors of m values from a sliding window moved over X. For each vector in the set, $x_{m}(i)$, the maximum element-wise distance (Chebyshev distance) is calculated. Let $B_{i}$ represent the number of vectors whose Chebyshev distance is ≤r. We then calculate the values:

(7) $$C_{i}^{m}(r)=B_{i}/(N-m+1)\phi^{m}(r)=(N-m+1{)}^{-1}\sum_{i=1}^{N-m+1}\text{ln}C_{i}^{m}(r)$$

Next, the steps are repeated for vectors of size m+1 and $A_{i}$, specified as the number of vectors with Chebyshev distance from $x_{m+1}(i)\leq r$. Finally, ApEn(m,r) is defined as ${\text{lim}}_{N\to \infty }\left[\phi^{m}(r)-\right.\left.\phi^{m+1}(r)\right]$, which is estimated as:

(8) $$\text{ApEn}(m,r,N)=\phi^{m}(r)-\phi^{m+1}(r)\approx (N-m{)}^{-1}\sum_{i=1}^{N-m}-\text{ln}\left(A_{i}/B_{i}\right)$$

The above statistic is typically approximated by only considering the range 1≤i≤N−m for the calculation of $A_{i}$ and $B_{i}$, thus excluding the vector at index i=N−m+1, and allowing the formula to be simplified as shown above.

### 4.3 Method 3: Sample Entropy (SampEn)

One drawback of ApEn is its bias on super short sequences that causes ApEn to skew to 0, sometimes incorrectly implying sequence regularity [14]. Richman and Moorman [2000] introduced Sample Entropy as a way to reduce this bias. The process of extracting vectors and calculating A(r) and B(r) is performed similarly to ApEn, except that the match of $x_{m}(i)$ with itself is not included in the totals, as it is with ApEn and instead of approximating the final value as shown in Equation 8, the value is calculated as the limit $\left({\text{lim}}_{N\to \infty }\right)$, of the negative logarithm of the ratio between $A^{m}(r)$ and $B^{m}(r)$ and the statistic SampEn(m, r, N) as:

(9) $$\text{SampEn}(m,r,N)=-\text{ln}\left[A^{m}(r)/B^{m}(r)\right]$$

## 5 CONVERGENCE ANALYSIS

Because we model a person’s frailty based on movement entropy, we are interested in determining how well the three proposed methods, USM-Rényi, ApEn, and SampEn, converge and distinguish sequences from distributions of different complexity. We conduct experiments to comparatively evaluate these methods using a Monte Carlo simulation of synthetic symbolic sequence data. All analyses are conducted using Python 3 and source code for USM functions can be found here https://doi.org/10.5281/zenodo.8180741. Other functions and data available on request.

We generate 10^3^ samples of independent and identically distributed (i.i.d.) uniformly random sequences of length 500 to 10000 for alphabet sizes ranging from 4 to 23. For each sample sequence, we compute ApEn and SampEn for each m=(1,2,3,4) with the tolerance r=0 as r is not needed when dealing with symbolic sequences [13]. We also compute USM coordinates and USM-Rényi entropy with the same kernel variance values, $\sigma^{2}$, used in [18]. To understand the precision of each entropy measure we estimate the standard error (SE) of its sampling distribution as the standard deviation of the entropy estimates for each sequence length and distribution.

The size of $\sigma^{2}$ in the USM-Rényi estimate roughly corresponds to the length k of k-gram density being estimated [18], therefore there is a congruence between $\sigma^{2}$ and the value of m used in ApEn and SampEn. Pairwise Pearson’s correlations between USM-Rényi, ApEn and SampEn for each value of $\sigma^{2}$ and m reveal that while for certain pairs of m and $\sigma^{2}$ there is strong correlation ( |r|≥0.9 for some pairs), the entropy measures are not one to one interchangeable and may each offer unique insight into the complexity of a sequence. However, analysis of the distributions and pairwise Pearson’s correlations of the USM-Rényi values reveals for $\sigma^{2}<1\times 10^{-7}\;SE\approx 0$ and for $\sigma^{2}\geq 0.1$ USM-Rényi estimates are completely correlated with each other (r=1). This implies no new information is gained regarding the complexity of the distribution of USM coordinates from kernel variances beyond these cutoffs and so we limit the remainder of our analyses to $\sigma^{2}$ values in the range $1\times 10^{-7}\leq \sigma^{2}\leq 0.1$.

For all entropy estimates, convergence rates are slower as the alphabet size, d, increases. For d=23 USM-Rényi values for each $\sigma^{2}$ converge for n≥2500 with small SE relative to the absolute values of the entropy measure. The absolute value of USM-Rényi is consistently greater for larger alphabet size for $1\times 10^{-}7\leq \sigma^{2}\leq 0.01$, though an inflection does occur at $0.0316<\sigma^{2}\leq 0.056$ where the signs of the estimates flip from negative to positive.

One downside to SampEn is it may return an undefined result when there are no m+1 length vector matches. In our simulations, the rate at which SampEn was undefined increased with alphabet size, such that, for d=23,65% and 19% of SampEn(m=3) values were undefined for n=500 and n=1000 respectively. And SampEn(m=4) was undefined for nearly all 500 and 1000 length sequences and was undefined for 65% and 16% of sequences length 2500 and 5000 respectively. For i.i.d. uniform distributions both ApEn and SampEn should converge to the same value for any m. Our results show this is the case for SampEn but not ApEn. ApEn(m=1) converges by n=2500 for d=23 but even for sequences length 10000, ApEn convergence is progressively worse for increasing m and d (Figure 3). Despite this slow convergence, for n≥2500 the spread of ApEn is very narrow with SE<0.01 for all m. SampEn, on the other hand, converges quickly for each value of m even with the smallest sample sizes but the SE for SampEn values is much larger than ApEn to the point of overlapping with other distributions (Figure 3a). For d=23, the SE of SampEn(m=2) for n=2500 is 0.06, nearly an order of magnitude larger than for ApEn(m=2) at n=2500(SE=0.008).

We also use simulated Markov Chains to gauge the ability of the entropy measures to distinguish between sequences with the same alphabet size but different probability distributions. We generate 10^3^ sequences of length n=5000 from two first-order Markov Chains with entropy rate, computed following the method in [19], of 1.059 and 1.308 respectively. All three entropy estimates correctly estimate the i.i.d. sequences to be the most complex and *MC1 A* as the least complex (Figure 3b). While all three methods have narrow enough SEs to distinguish between the distributions, the relative range of ApEn and SampEn is much smaller than USM-Rényi.

## 6 SMART HOME CASE STUDY

We highlight the use of USM to analyze the complexity of smart home behavior data through a case study. To illustrate the analysis, we use a technique proposed by Almeida and Vinga [3] to generate 2D representations of USMs. In this visualization, each sensor in the home is assigned to one vertex in an equilateral polygon. The density of points plotted by the USM algorithm creates a fractal-like pattern of sub-polygons (i.e. sub-quadrants in the 4d version shown in Figure 2) representing multi-order transitions between locations. We analyze maps (Figure 4) generated from three days of data from 13 smart home motion detectors placed around the home of an 83-year-old female resident. For comparison, Figure 4a demonstrates how synthetic uniform random data for this smart home configuration generates a map with fully filled nested polygon rings.

The remaining plots in Figure 4 represent three different days of (self-reported) activity: a normal day, a day with housekeeping activities, and a day with a visitor in the home. On the normal day, we observe that the most complete rings occur within a room, with occasional transitions between bedroom and bathroom, living room and sink, and entryway and living room. Overall, transitions between distinct areas of the home are less common.

In contrast, the days with housekeeping and visitor activities generate darker, more complete rings. Figure 4c shows the distribution of motion is fairly uniform for the housekeeping day, while the visitor day highlights more frequent multi-order transitions in and between the living room, kitchen, and dining area. The USM maps align with the reported activities of the participant, visualizing both the amount and diversity of movement within the space, including transitions between regions. This visualization provides a valuable foundation for analyzing movement complexity as an indicator of frailty.

## 7 DISCUSSION AND CONCLUSION

In this paper, we introduce and compare entropy estimates of smart home-based human behavior as a foundation for analyzing frailty.

In addition to the popular approximate and sample entropy measures, we also consider universal sequence maps for analyzing such data. We provide a proof of USM based Rényi entropy for any alphabet size. This is a useful advance as USMs provide a simple method to compute the multi-order transition frequencies of smart home behaviors which can be described by an arbitrary number of ambient sensors. USMs also provide a valuable method for visualizing behavior complexity from collected ambient sensor readings.

Our analysis shows that these entropy measures are very dependent on both sequence length and alphabet size. While all methods converge on simulation data, faster convergence also sometimes coexisted with larger error. Researchers should exercise caution when drawing conclusions related to differences in entropy of smart home data if the sequences are very short or of different lengths, or if they come from smart homes with different sensor counts. Of the methods we analyzed, USM-Rényi showed a favorable balance of convergence rate, precision, and relative consistency across different alphabets and distributions compared to ApEn and SampEn.

A limitation of the work is that analysis is based on simulation data. We conjecture that behavior complexity, estimated by entropy of ambient sensor data, is an indicator of frailty. Further work is needed to validate this based on clinical assessment of subject frailty and how it compares with other behavior features such as total within-room and between-room movement. Additionally, our visualizations reflect periods of increased complexity that are due to visitors in the home. Analysis of frailty will need to control for such cases to analyze behavior that is based on one specific person in the home. This work provides a new method to model behavior complexity from smart home sensor data, capturing temporal movement patterns and creating a foundation for passive, continuous in-home health assessment.

## Figures and Tables

**Figure 1:**
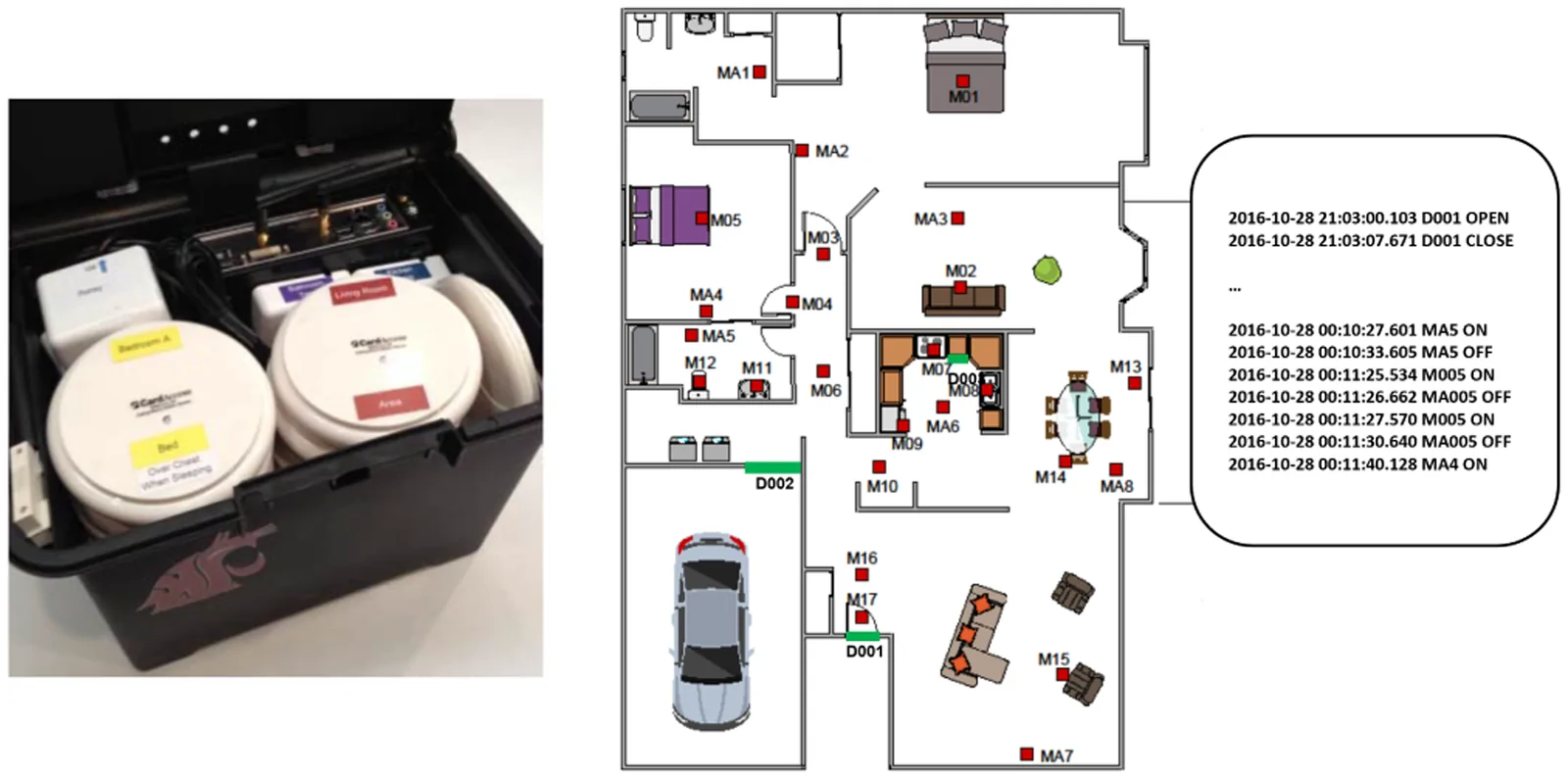
CASAS SHiB. Infrared motion detectors are installed inside the refrigerator and in each functional area of the house. Magnetic door closure sensors are attached to external doors and selected cabinets. Sensor readings are stored by the smart home for analysis.

**Figure 2:**
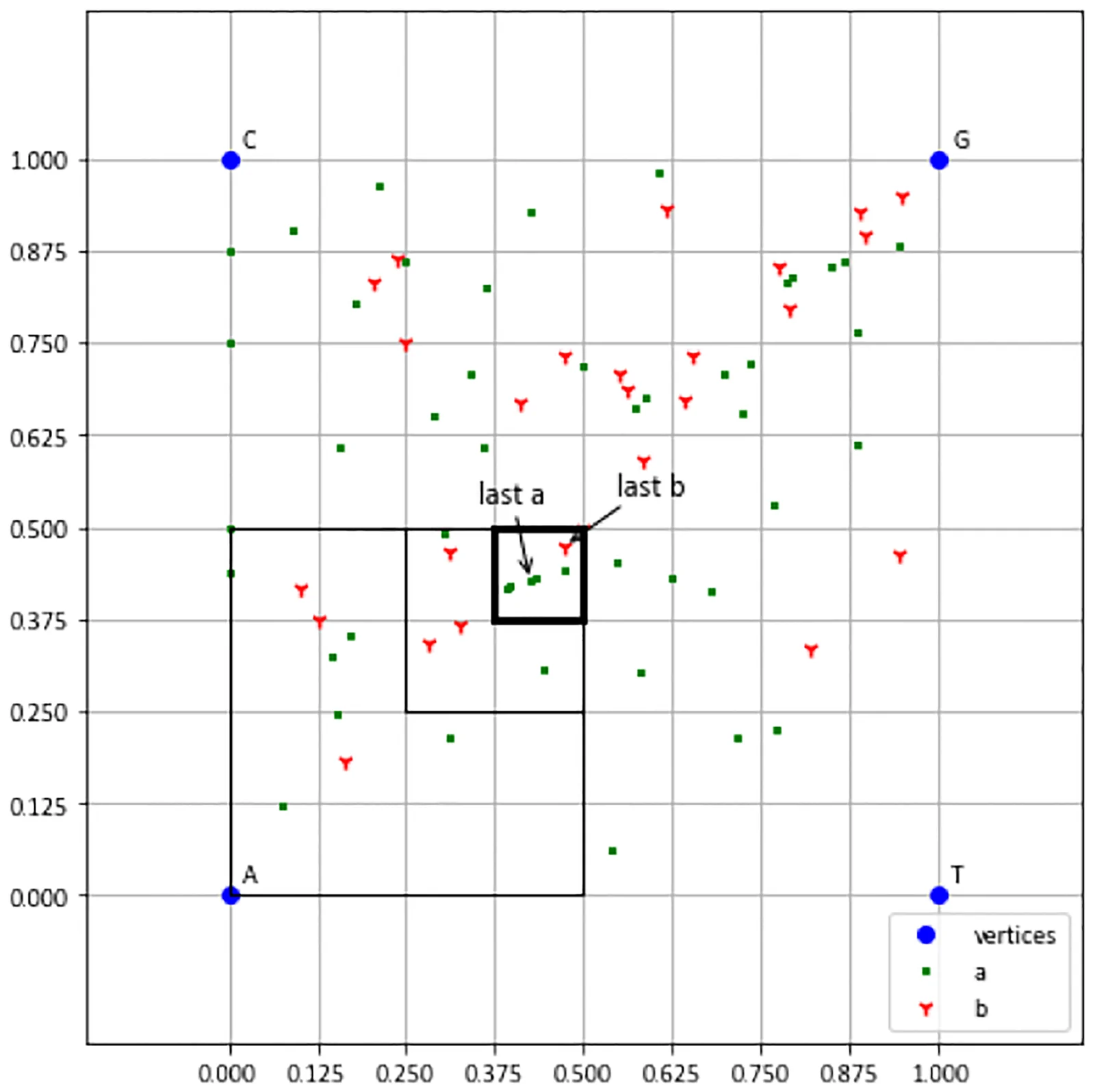
CGR plot of sequences with a shared suffix. The last 4 symbols of a are ‘AGGA’ and the last 4 of b are ‘GGGA’. The highlighted sub-quadrant contains the coordinate of the sequences’ last symbol, the quadrant size corresponds to the suffix length.

**Figure 3:**
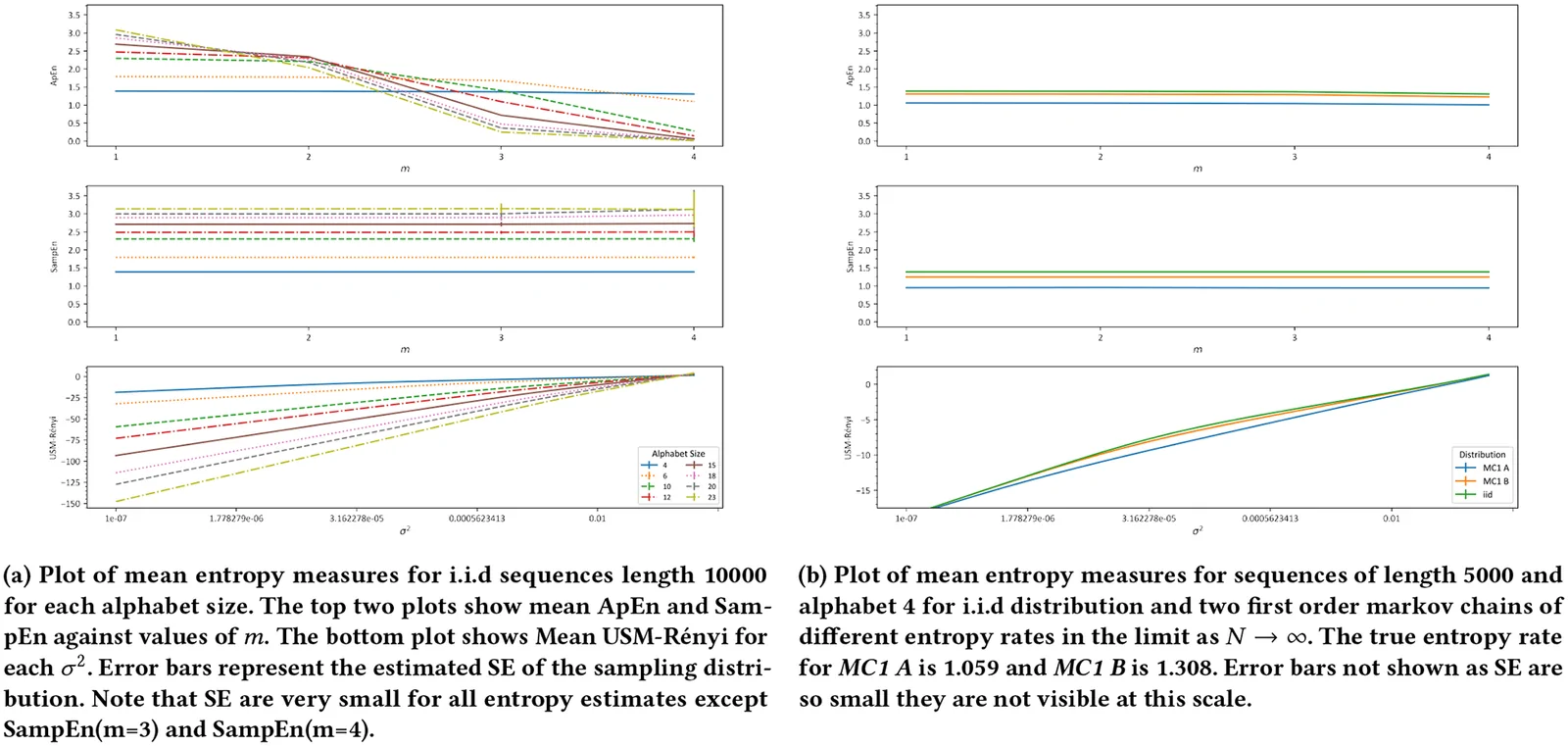
Ability of entropy estimates to distinguish sequences with different underlying distributions.

**Figure 4:**
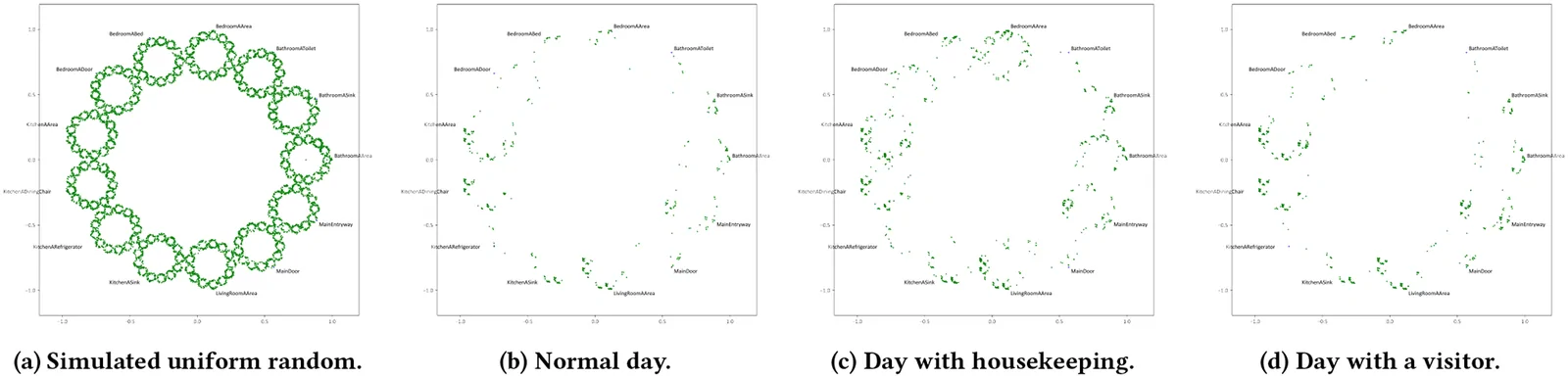
USM plots for 13 motion sensors in a smart home. The completeness of nested sub-polygons indicates the relative frequency of transitions from other locations.

## CCS CONCEPTS

• General and reference → Experimentation; • Mathematics of computing → Information theory; *Markov processes*; Time series analysis; • Human-centered computing → Empirical studies in ubiquitous and mobile computing.
